# Identification of a Novel Luminal Molecular Subtype of Breast Cancer

**DOI:** 10.1371/journal.pone.0103514

**Published:** 2014-07-30

**Authors:** Anna Dvorkin-Gheva, John A. Hassell

**Affiliations:** Centre for Functional Genomics, Department of Biochemistry and Biomedical Sciences, McMaster University, Hamilton, Ontario, Canada; The Norwegian University of Science and Technology (NTNU), Norway

## Abstract

The molecular classification of human breast tumors has afforded insights into subtype specific biological processes, patient prognosis and response to therapies. However, using current methods roughly one quarter of breast tumors cannot be classified into one or another molecular subtype. To explore the possibility that the unclassifiable samples might comprise one or more novel subtypes we employed a collection of publically available breast tumor datasets with accompanying clinical information to assemble 1,593 transcript profiles: 25% of these samples could not be assigned to one of the current molecular subtypes of breast cancer. All of the unclassifiable samples could be grouped into a new molecular subtype, which we termed “luminal-like”. We also identified the luminal-like subtype in an independent collection of tumor samples (NKI295). We found that patients harboring tumors of the luminal-like subtype have a better prognosis than those with basal-like breast cancer, a similar prognosis to those with ERBB2+, luminal B or claudin-low tumors, but a worse prognosis than patients with luminal A or normal-like breast tumors. Our findings suggest the occurrence of another molecular subtype of breast cancer that accounts for the vast majority of previously unclassifiable breast tumors.

## Introduction

The cellular heterogeneity and genetic complexity of breast carcinomas cannot be completely captured using only clinico-pathological tumor characteristics [1], [2], [3]. The discovery of various molecular subtypes of human breast tumors has led to a better understanding of their biology and has had an impact on breast cancer patient prognosis and clinical care. However, a relatively large fraction of breast tumors approaching 35% in some collections cannot be assigned to one or another molecular subtype [3]. The goal of our study was to determine whether these unclassifiable tumors comprise one or more previously un-described molecular subtype(s).

Studies performed using global gene expression profiling introduced molecular classification of breast tumors based on ∼500 ‘intrinsic’ genes known to exhibit high variability of expression between different groups of tumors, but to have a stable level of expression in the same tumor over time and/or after one or another therapy [1]. Based on this molecular taxonomy, breast tumors were broadly divided into 2 groups contingent on their expression of the estrogen receptor (ESR1, hereafter termed ER). These 2 groups could be further subdivided into 5 subgroups: 2 ER+ (luminal A and luminal B) and 3 ER- (basal-like, ERBB2-overexpressing (ERBB2+), and normal-like). More recent studies revealed 2 additional albeit less common subtypes termed ‘claudin-low’ and ‘molecular apocrine’ (mApo; [4], [5], [6], [7], [8]).

Tumors of the luminal A and luminal B subtypes are distinguished based on their relative expression of the ER, ER-regulated genes, the progesterone receptor (PR) and other genes such as GATA-binding protein 3, X-binding protein and hepatocyte nuclear factor 3 alpha, which are also expressed by normal breast luminal cells [1], [2]. Tumors of the ERBB2-overexpressing molecular subtype express high levels of ERBB2 and other genes such as *GRB7* located on the 17q ERBB2 amplicon [1], [2]. The basal-like molecular subtype tumors do not express the ER, the PR or overexpress ERBB2, but express cytokeratins (CK) 5, and/or CK 17, which are also expressed by the basal myoepithelial cell layer of the normal breast epithelium [1], [2]. The normal-like subtype tumors express genes commonly attributed to adipose tissue [1], [2]. The claudin-low molecular subtype tumors are characterized by low expression of Claudins 3, 4, and 7, E-Cadherin and CD24 [7], [8], whereas apocrine molecular subtype tumors typically do not express the ER but express the androgen receptor (AR) at high levels [5], [6].

All breast tumor samples cannot be assigned to one or another molecular subtype. For example an early study examining 3 independent collections of gene expression profiles of human breast tumors comprising 115, 117 and 49 samples revealed that 35.2%, 25.8% and 6.1% respectively of these specimens could not be assigned to one of the known molecular subtypes [3]. Samples that cannot be assigned to a molecular subtype are usually not included in subsequent studies that involve or are based on any features of molecular subtypes.

Because the unclassifiable breast tumor samples comprise a high fraction of all tumors and could potentially comprise novel molecular subtypes, we investigated means of classifying these samples. To this end we used a collection of publically available gene expression profiles obtained from 1,593 human breast tumor samples. We could not assign 24.9% of these samples to any of the 7 identified molecular subtypes. We subsequently discovered that all of these unclassified samples comprised a new molecular subtype, which we termed “luminal-like”. Luminal-like subtype tumors are characterized by increased interferon alpha/beta signaling, and a decrease in metabolic processes, ERBB2 signaling and integrin cell-surface interactions. Patients with tumors of the luminal-like subtype are predicted to have a better prognosis than those who experienced basal-like breast cancer, a similar prognosis to those with ERBB2+, luminal B, or claudin-low tumors, but a worse prognosis than patients with luminal A and normal-like breast tumors.

## Methods

### Data collection

Patient gene expression profiles and accompanying clinical data was publically available and as such their use did not require local ethics board approval. In the course of our study we analyzed the gene expression profiles *in silico* of 7 publically available datasets, obtained using Affymetrix HG-U133A GeneChip arrays. These profiles were deposited in the Gene Expression Omnibus (GEO) (accession numbers GSE3494, GSE1456, GSE7390, GSE2034, GSE6532, GSE17705 and GSE25066) and comprise a total of 2,027 samples. All the samples are accompanied with clinical follow-up data. However, because some of the datasets were contributed by the same source, there were redundant samples in several datasets. Hence these redundant sample profiles were removed thus reducing the number of unique samples to 1,695. All samples used for our study were normalized with frozen Robust Multi-array Analysis (fRMA), a procedure that allows one to pre-process microarrays individually or in small batches and to then combine the data into a single dataset for further analysis [9]. We then used the DWD (Distance-Weighted Discrimination, [10]) and ComBat [11] methods to remove technical variation from the datasets that were to be combined for future analysis. After combining all datasets Pearson correlation coefficients for pair-wise comparisons of samples using 68 house-keeping probe sets were computed, and only samples exhibiting a correlation higher than 0.95 with at least half of the dataset were selected for further classification. The latter filtering method yielded a dataset comprising 1,593 human breast tumor sample transcript profiles.

### Molecular subtype assignment

Samples were classified as basal-like, ERBB2+, luminal A, luminal B, claudin-low, normal-like or apocrine by assigning them to a cluster representing that subtype to a standardized centroid of which they had the highest Spearman rank correlation [1], [3], [5], [7]. The correlation was computed using the 710 intrinsic genes. The intrinsic gene list used to define basal-like, luminal A, luminal B, ERBB2-overexpressing, and normal-like molecular subtypes comprises 496 genes [1]. However, in subsequent studies that identified the claudin-low subtype, a larger set of 1,918 intrinsic genes was used [12]. Similarly the apocrine subtype was defined by using 3,198 genes [5]. To perform subtype assignments among our collection of breast tumor profiles we used 710 intrinsic genes (see Table S1) that were shared between the sets of 1,918 and of 3,198 genes.

The standardized centroid was computed for each subtype as follows: the average expression of each gene across the subtype was divided by the standard deviation of expression of that gene across that subtype. Reference samples used to calculate standardized centroids for the apocrine subtype were taken from Farmer et al [5] and for the basal-like, ERBB2+, luminal A, luminal B, claudin-low and normal-like subtypes from Prat et al ([8]; https://genome.unc.edu/pubsup/clow/UNC337arraydata_imputedCollapsedannotation.txt). Expression profiles obtained from the Farmer study [5] were composed of 3 molecular subtypes: apocrine, luminal and basal-like. After using the DWD procedure to remove technical variation among our sample collection and the other reference samples, we verified that the luminal and basal-like samples obtained from the Farmer study clustered with luminal A and B, and basal-like samples as identified by Prat et al [8] respectively. This comparison indicated that technical variation was removed sufficiently to use the centroid for the apocrine subtype together with the centroids for the other subtypes to assign subtypes to the curated 1,593 breast tumor samples. Gene symbols were used to match the probes and genes with Gene Symbol names. These data were averaged and samples were median-centered for all datasets prior to subtype assignment.

PAM50 [12] has also been widely used for breast tumor classifications. Therefore, to validate our method of classification, we also compared our classification results with those obtained using PAM50. PAM50 was performed by using the ‘pamr’ package in R. Because PAM50 classifies samples into only 5 subtypes (basal, ERBB2+, luminal A, luminal B and normal-like), we compared the classification results for these subtypes while validating our method. We compared samples classified by both methods to identify the fraction of samples that were classified similarly. We found these fractions to be significantly high for all the examined subtypes (Basal: p-value<0.000001, ERBB2+: p-value<0.000001, Luminal A: p-value<0.000001, Luminal B: p-value<0.000001, Normal-like: p-value<0.000001; all p-values were corrected with Bonferroni correction for multiple testing). This finding supported our method for molecular subtype classification and provided the basis for further analyses.

### Reproducibility of molecular subtype clusters

The reproducibility of clusters 1 and 2 (described in Results), and that of the luminal-like cluster (Results) was examined by using the IGP (In-Group Proportion) metric [13] with an independent NKI dataset comprising 295 human breast tumors [14] that was downloaded from http://www.rii.com/publications/2002/default.html. This dataset was combined with the reference samples from Prat et al [8] and Farmer et al [5] by using 5,688 genes shared among all the samples. Technical variation was removed by using DWD. Thereafter, the basal-like, ERBB2+, luminal A, luminal B, claudin-low, normal-like and apocrine standardized centroids were computed for all 5,688 genes from the reference samples. Among the 710 intrinsic genes (see “Molecular subtype assignment” section), 518 genes were shared by all samples (see Table S2) and were used for the IGP. We used the “ReproCluster” package (Bioconductor; [13]) to calculate the IGP for all NKI samples in relation to the normalized centroids calculated from the reference samples.

### ER and PR status of samples

Because the majority of the samples did not have information about their ER and PR protein status, we established the ER and PR status of samples by using our previously published gene signatures predictive of ER and PR status [15]. The signature predictive of ER status consists of 24 genes and that predictive of PR status comprises 51 genes.

### Luminal C subtype

Because the raw data used to build a standardized centroid for the luminal C subtype was not available, we used information reported in the study that defined this subtype [2]. Based on latter study, the luminal C subtype exhibited 2 clusters of highly expressed genes: luminal cluster G (highly expressed in all luminal subtypes) and cluster D (highly expressed in luminal C, basal-like, and ERBB2+ subtypes, but not in the luminal A or luminal B subtypes). Cluster D comprises 11 genes an EST (AA010188), *TFRC* (N21329), *MYBL2* (AA456878), *KIF23* (AA452513), *LAPTM4B* (AA600214, AA033947), *GGH* (AA455800), *FJL10511* (AA115275), *YBX1* (AA599175), *EBNA1BP2* (T74979), *YWHAZ* (AA609598), and *SQLE* (R01118)). Two genes (EST and hypothetical protein FJL10511) were not represented on the arrays used in our study; therefore, 9 of 11 genes were used for our analysis. Cluster G comprises 13 genes/hypothetical proteins including GPR160 (H50224), *ACADS*B (H95792), *ESR1* (AA291749), *TFF3* (N74131), *GATA3* (R31441, H72474), *XBP1* (W90128), *FOXA1* (T74639), *AFF3* (H99588), *LIV1* (H29315), *NPNT* (AA029948), *TUBA1C* (N54508), *NAT1* (R91802), *MYO6* (AA625890, AA030004). Two of these genes (*GPR160* and *NPNT*) were not represented by probe sets on the arrays used in our study; therefore, 11 genes were used for further analysis. We used these 20 genes to determine whether the transcript profiles of the unclassified samples could be assigned to the luminal C subtype by performing hierarchical clustering (Spearman distance, Average linkage) and examining whether these genes were up-regulated in samples of the luminal-like cluster.

### Survival analysis

To perform survival analysis we used the “survival” package (Bioconductor; http://cran.r-project.org/web/packages/survival/index.html). The analysis included the “disease-free” and “overall” survival parameters. Because clinical information supplied with the breast tumor samples did not always include both clinical parameters, 928 and 963 among the 1,593 samples were available for disease-free and overall survival analyses respectively.

The following pair-wise comparisons were performed: survival parameters of patients with tumors assigned to the luminal-like subtype were compared to parameters of patients with tumors assigned to each of the other 7 molecular subtypes. Bonferroni correction was applied on the p-values obtained from all the pair-wise comparisons.

### Differential gene expression analysis

We used the “limma” package (Bioconductor; [16]) for comparing profiles of samples assigned to the luminal-like subtype to those of the other subtypes (7 pair-wise comparisons in total). To this end the moderated F-statistic was used, followed by Benjamini-Yekutieli adjustment for multiple testing [17]. Only genes differentially expressed with at least a 2-fold change were examined and analyzed further.

### Pathway analysis

We did not identify any genes whose expression was unique to tumors of the luminal-like subtype. Each of the differentially expressed genes identified in the pair-wise comparisons followed one of three patterns: (1) genes that were expressed at their highest level in the luminal-like and another subtype(s) compared to the remaining subtypes; (2) genes that were expressed at their lowest level in the luminal-like and another subtype(s) compared to the remaining subtypes; and (3) genes that were expressed at an intermediate level in the luminal-like and another subtype(s) compared to the remaining subtypes.

We selected genes for pathway analysis representative of patterns 1 and 2 described above. A complete list of these genes is shown in Table S3. The pathway analysis was performed with Reactome [18]; lists of up- and down-regulated pathways were obtained and p-values were adjusted with Benjamini-Yekutieli adjustment for multiple testing [17].

## Results

### Identification of a novel reproducible subtype

**Figure 1:**
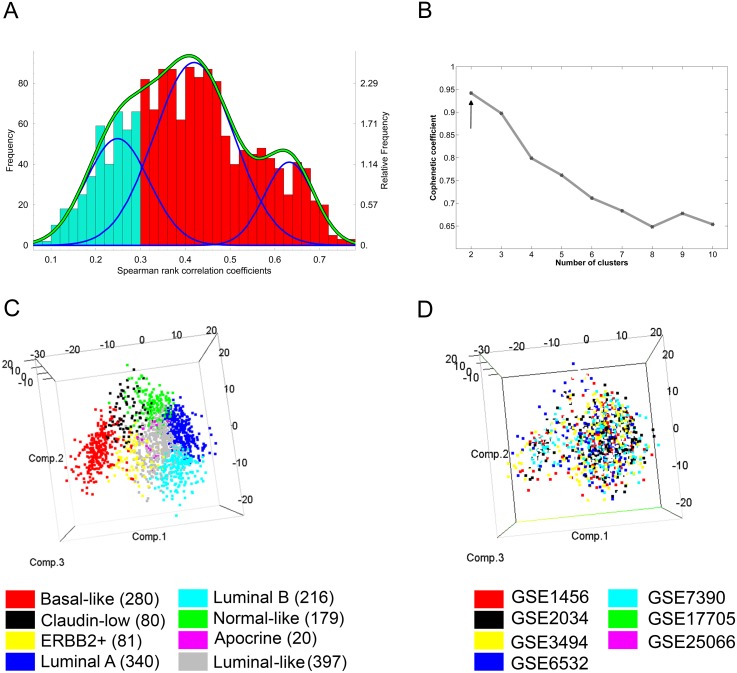
Identification of the novel cluster. A. Distribution of Spearman Rank Correlation Coefficients. Distribution of correlation coefficients is shown in the form of a histogram. Gaussian distributions fitted by using the EM (Expectation-Maximalization) algorithm are shown in blue, the sum of these distributions is shown in green. Correlation coefficients lower than than 0.3 are marked in cyan; correlation coefficients higher than 0.3 are marked in red. B. Cophenetic coefficient obtained from NMF clustering. Optimal number of clusters established by this method is indicated by black arrow. C. Principal Component Analysis performed on 1,593 tumor samples by using 710 genes (see Table S1). Samples are colored by the subtype they were assigned to. D. Principal Component Analysis performed on 1,593 tumor samples by using 710 genes (see Table S1). Samples are colored by the dataset of their origin.

As reported previously, subtype assignment is performed by identifying a centroid representing a subtype to which a sample has the highest correlation [3]. The latter study did not assign subtypes to samples with a maximal correlation coefficient lower than 0.1, assuming that such low coefficients indicate that the samples are not similar enough to any of the examined subtypes and therefore cannot be assigned to any of them. However, because the cutoff was chosen based on a relatively small collection of samples and because the collection of samples we assembled for our study was much larger than that used initially by Perou and colleagues [1], [3], we attempted to re-define the cutoff. To the latter end we re-examined the distribution of correlation coefficients (Figure 1A) by using the EM (Expectation-Maximalization) algorithm [19] and found a statistically significant cutoff (p = 0.0018) of 0.3 for these coefficients. Hence the latter cutoff was used for subtype assignment.

When attempting to assign molecular subtypes to 1,593 publically available human breast tumor global transcript profiles we found that 397 samples could not be assigned to one or another molecular subtype because they had a low correlation (Spearman rank correlation coefficient <0.3) to the standardized centroids previously established for each of the 7 molecular subtypes (see cyan bars in Figure 1A). To determine the number of clusters present in the population represented by these 397 unclassifiable samples, we used the cophenetic coefficient obtained by Nonnegative Matrix Factorization (NMF) clustering [20]. The data in Figure 1B show that, based on NMF clustering, the optimal number of clusters was two. However, two is the minimal number of clusters that can be identified by the NMF procedure. In consequence we also explored the possibility that the unclassifiable samples might comprise a single cluster.

We used an independent dataset of 295 human breast tumors (NKI; [14]) to verify that the two clusters defined by the NMF procedure were reproducible. To this end we used the In-Group Proportion (IGP) measure [13], which addresses cluster reproducibility in various datasets. IGP showed that only one of the two clusters was reproducible (p-value = 0.03; ‘clusterRepro’ package). However, when both clusters were combined into a single cluster, it was found to be reproducible in the NKI dataset (IGP; p-value = 0.012; ‘clusterRepro’ package). The latter finding provided support for the existence of a single cluster comprising all 397 samples that could not be assigned to any of the existing 7 molecular subtypes. Therefore, our further work concentrated on a single candidate cluster composed of these 397 “unclassifiable” samples.

To confirm that the subtype classification separated tumor samples into homogeneous groups we performed Principal Component Analysis (‘rgl’ package, R) with 710 genes used for the classification of the samples (Figure 1C). As can be seen, the samples clearly segregated based on their assigned subtype. Additionally, we verified, that there were no cohort combination artifacts by examining the distribution of samples from separate data sets. Figure 1D shows, that samples from all datasets were equally dispersed across the 3 dimensional space as defined by the Principal Component Analysis.

### Relationship between unclassifiable samples and the luminal A and B molecular subtypes

To examine the relatedness of the candidate luminal-like molecular subtype to those identified previously, we performed hierarchical clustering of the standardized centroids for all of the subtypes. Centroids for the established subtypes (basal-like, claudin-low, ERBB2+, luminal A, luminal B and normal-like) and the mApo subtype were computed based on reference samples from Prat [8], and Farmer [5] respectively, whereas the centroid for the luminal-like subtype was calculated based on the 397 samples defined as luminal-like in our tumor collection. The candidate luminal-like subtype seemed to be related to the luminal A and luminal B subtypes (see “luminal-like” centroid in Figure S1). The latter observation was also supported by examining the Spearman rank correlation coefficients between the standardized centroids representing the candidate luminal-like subtype and the other molecular subtypes (Table 1). The luminal-like subtype displayed the highest correlation to the luminal B subtype and the next highest correlation to the luminal A subtype (0.47 and 0.38 respectively). Additional evidence for a high correlation among the 397 “unclassifiable” samples, and the luminal A and B subtypes came from the fact that 378 of 397 (95.2%) luminal-like samples were found to be ER and/or PR positive. Hence we termed the new subtype “luminal-like” to reflect its similarity to the other luminal molecular subtypes.

**Table 1:** Spearman rank pair-wise correlation coefficients between the 7 subtypes and Luminal-like.

	Basal	Claudin-low	ERBB2+	Luminal A	Luminal B	Normal-like	mApo	Luminal-like
**Basal**	*1.00*	0.27	0.28	−0.74	−0.44	−0.01	−0.11	−0.23
**Claudin-low**		*1.00*	−0.01	−0.38	−0.50	0.32	−0.12	0.04
**ERBB2+**			*1.00*	−0.28	0.07	−0.19	0.49	0.19
**Luminal A**				*1.00*	0.53	0.16	0.08	0.38
**Luminal B**					*1.00*	−0.49	0.04	**0.47**
**Normal-like**						*1.00*	0.07	−0.02
**mApo**							*1.00*	0.10
**Luminal-like**								*1.00*

The highest correlation between Luminal-like and any of the other subtypes is marked in Bold. The correlation of a centroid to itself is marked in Italics.

**Figure 2:**
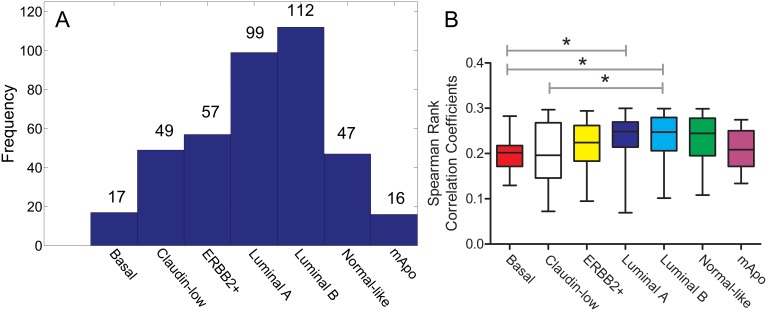
Correlation of 397 unclassified samples with 7 subtypes. A. Distribution of samples based on highest correlation coefficient (excluding the correlation to “Luminal-like” centroid) B. Comparison of the same correlation coefficients across subtypes. The basal-like subtype showed lower coefficients than the luminal A and luminal B subtypes and claudin-low subtype showed lower coefficients than luminal B subtype (Kruskal-Wallis (p-value = 0.0004, post-hoc Dunn’s multiple comparison test)).

We wondered whether the luminal-like subtype was an artifact, resulting from the fact that the luminal A and luminal B standardized centroids do not reflect the variability of all samples that comprise these two subtypes. Hence we tested whether the 397 samples could be classified as either luminal A or luminal B if a correlation coefficient of less than 0.3 was used. As shown in Figure 2A in the absence of a luminal-like centroid, only 53% of samples would have been assigned to either the luminal A or luminal B subtypes, if correlation coefficients cutoff of less than 0.3 was used (for full list of samples and their assigned subtypes, see Table S4). The latter finding indicates that the gene expression profiles of nearly half of the unclassifiable samples had a lower correlation to the luminal A and B subtypes, than they did to the other subtypes, and therefore could not be assigned to either the luminal A or B subtypes, yet possessed luminal characteristics.

We subsequently compared the maximal correlation coefficients used for the subtype assignment of the 397 luminal-like samples across all subtypes. Only the basal and claudin-low subtypes displayed lower correlation coefficients than did the luminal subtypes to the luminal-like subtype (Figure 2B). Hence the samples that might have been classified as luminal A or B if a correlation coefficient cutoff of less than 0.3 was used, did not cluster more closely to the luminal A and B centroids than the rest of luminal-like samples did to the other centroids. Based on these findings we conclude that although the luminal-like samples are related to the luminal A and B subtypes, they are not an artifact of the classification process. Moreover, the luminal-like samples are not members of either the luminal A or B subtypes, and likely comprise a novel molecular subtype.

### Relationship between the luminal-like and luminal C subtypes

A luminal C molecular subtype has previously been reported [2]. Whereas this subtype was not reproduced in subsequent studies [3], [8], [12], we attempted to determine whether there was a relationship between the unclassifiable samples, which we defined as luminal-like, and the luminal C subtype samples. We used the information reported by Sorlie et al [2], specifically the fact that samples of the luminal C subtype exhibited high expression of 2 gene clusters: luminal cluster G genes, whose expression is high in all luminal subtypes ([2]), and another gene cluster (Cluster D), which was not expressed in the luminal A or B subtypes, but was expressed in basal-like, ERBB2+ and luminal C subtype tumors [2].

**Figure 3:**
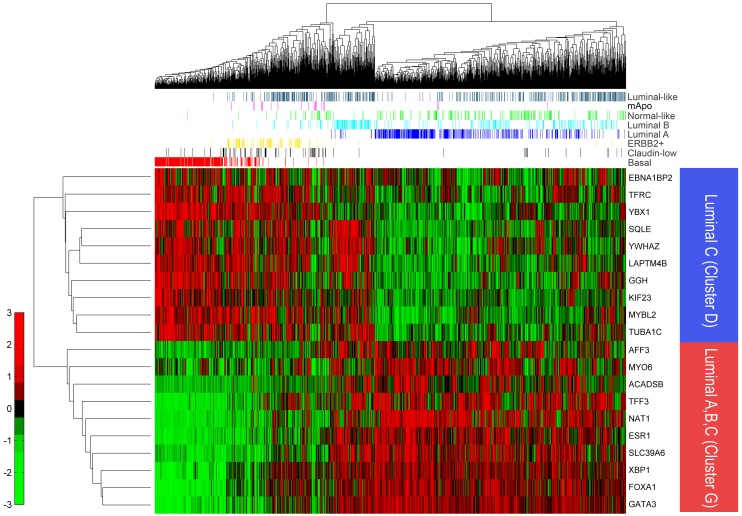
Hierarchical clustering of 1,593 samples based on genes belonging to Clusters D and G. Clustering was performed by using Spearman (columns) and Euclidean (rows) distance and average linkage metrics for both genes and samples. Genes from Clusters D and G are marked by blue and red respectively. Each sample belongs to one of the 8 molecular subtypes and is marked by a bar, colored based on the subtype: basal (red), claudin-low (black), ERBB2+ (yellow), luminal A (blue), luminal B (cyan), normal-like (green), molecular Apocrine (mApo; magenta), luminal-like (dark gray).

As shown in Figure 3, the luminal-like subtype was not characterized by high expression of the genes comprising clusters D and G, suggesting that the luminal-like subtype does not possess characteristics of the luminal C subtype and likely represents a unique molecular subtype.

### Survival analysis

Patients who harbored tumors of one or another molecular subtype have different prognoses [2], [14]. For example, patients who experienced basal-like or ERBB2+ breast cancer have a worse prognosis compared to those that were identified with luminal A or normal-like breast tumors. Hence we wondered whether patients who had experienced luminal-like subtype tumors had a better or worse prognosis than those who had tumors of each of the other molecular subtypes. The clinical measures we used for such comparisons were “disease-free survival” and “overall survival”, which were obtained from the supplementary information accompanying the breast tumor samples.

**Figure 4:**
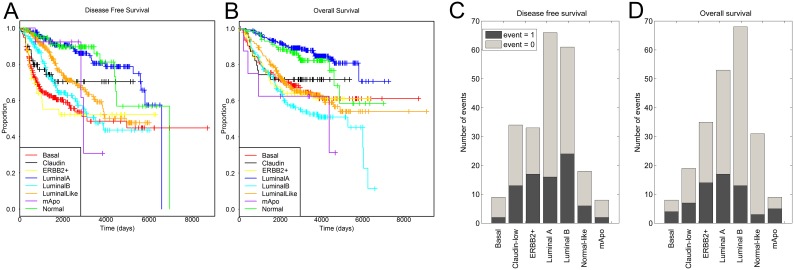
Kaplan-Meyer curves of survival rates across molecular subtypes. A. Disease-free survival rates (p-value = 0.000000000000784, Log-Rank test). B. Overall survival rates (p-value = 0.00000000000647, Log-Rank test). C. Distribution of disease free survival events across molecular subtypes, if luminal-like tumors were assigned into other subtypes. Number of samples patients with good (event = 0) and poor (event = 1) outcome assigned into each subtype. D. Distribution of overall survival events across molecular subtypes, if luminal-like tumors were assigned into other subtypes. Number of samples patients with good (event = 0) and poor (event = 1) outcome assigned into each subtype.

As shown in Figure 4A&B and Table 2, patients with tumors of the luminal-like subtype had a better prognosis than those with basal-like breast cancer, a similar prognosis to those with ERBB2+, luminal B or claudin-low tumors, but a worse prognosis than patients with luminal A and normal-like breast tumors. For example, disease-free survival of patients with luminal-like, claudin-low, ERBB2+ and luminal B tumors at 5 years was between 52.2% and 76.8%, whereas the same metric for patients with basal-like tumors was 60.4%, and for patients with luminal A and normal-like tumors it was at 90.6% and 89.6%, respectively. Overall survival at 5 years for patients with luminal-like, basal-like, claudin-low, ERBB2+, and luminal B tumors varied between 66.9% and 76.4%, whereas the same metric for patients with luminal A and normal-like tumors was at 92.9% and 89.4%, respectively.

**Table 2:** Disease-free (DFS) and overall survival (OS) of patients with tumors belonging to Luminal-like subtype compared to other subtypes.

Subtype	Total numberof samples	Number ofsampleswith DFSinformation	Number of sampleswith OSinformation	Corrected p-values vs. Luminal-like (Log-rank test, Bonferroni correction)		Survival at 5 years	
				DFS	OS	DFS	OS
**Basal**	280	204	125	**0.0002618 [HR: 0.507** **CI95%: 0.347–0.683]**	1	**60.4**	72.1
Claudin-low	80	50	39	1	1	70.4	71.8
ERBB2+	81	40	64	0.2422000	1	52.2	67.6
**Luminal A**	340	179	246	**0.0002639 [HR: 2.4** **CI95%: 1.584–3.578]**	**0.0000000655 [HR: 3.044** **CI95%: 2.052–4.303]**	**90.6**	**92.9**
Luminal B	216	124	135	0.3759000	0.233	64.3	66.9
**Normal-like**	179	95	116	**0.0159600 [HR: 2.313** **CI95%: 1.309–3.309]**	**0.00659 [HR: 2.202** **CI95%: 1.318–2.963]**	**89.6**	**89.4**
mApo	20	13	9	1	1	92.3	62.5
Luminal-like	397	223	229	na	na	76.8	76.4

Comparisons with corrected p-values<0.05 are marked in bold and Hazard Ratios (HR) and their 95% Confidence Intervals are provided in brackets.

The finding that the survival rate of patients with luminal-like tumors is different than that of patients with tumors of some of the other subtypes could represent the fact that had the tumors from patients with a good prognosis not been classified into the luminal-like subtype, then they would have been assigned to either the luminal A or normal-like subtype, whereas tumors from the patients with poor prognosis would have been assigned to either the basal, luminal B or ERBB2+ subtypes. To explore this potential, we examined the prognosis of patients with luminal-like tumors across the subtypes the tumors would have been assigned had they not been assigned to the luminal-like subtype (please see Figure 2A for the distribution of these patients into other subtypes).

Figure 4C&D shows that there was no specific prevalence of patients with poor prognosis among any of the subtypes for either disease-free survival (left) or overall survival (right). These data suggest that patients with poor prognosis do not necessarily bear tumors that have characteristics of molecular subtypes with poor outcome, such as basal, luminal B and ERBB2+, and patients with good prognosis do not bear tumors characteristic of molecular subtypes with good outcome, such as luminal A and normal-like subtypes. Therefore, the duality of patient survival is not a reflection of distribution of the 397 unclassifiable samples across the previously established molecular subtypes.

### Genes differentially expressed between luminal-like and other molecular subtypes

To determine whether tumors classified as luminal-like are characterized by different biological processes, we performed pair-wise differential gene expression analyses between luminal-like tumors and those of the other molecular subtypes. Comparison of the transcripts expressed between luminal-like subgroup samples and those of each of the other molecular subtypes identified differentially expressed genes with a fold change of 2 or greater (Table 3; for a detailed gene lists see Tables S5 and S6). Only 14 and 8 genes were differentially expressed between luminal-like and luminal A and B tumors respectively. The greatest number of differentially expressed genes was found between the luminal-like and the basal-like and molecular apocrine subtypes (164 and 160 respectively), suggesting that luminal-like subtype tumors are least related to these two subtypes.

**Table 3:** Genes differentially expressed between Luminal-like and the other molecular subtypes.

	11,982 genes (710 intrinsic genes)	
	↓	↑
**Vs. Basal**	74 (44)	90 (51)
**Vs. Claudin-low**	48 (10)	51 (32)
**Vs. ERBB2+**	30 (14)	19 (13)
**Vs. LumA**	12 (8)	2 (2)
**Vs. LumB**	**6 (5)**	**2 (1)**
**Vs. Normal-like**	48 (26)	13 (9)
**Vs. mApo**	111 (42)	49 (27)

Down-regulated genes are marked by “↓”, up-regulated – by “↑”. Numbers in brackets indicate number of differentially expressed genes out of the 710 intrinsic genes used for subtype assignment. Lowest numbers of differentially expressed genes across subtypes are marked in Bold.

We used the differentially expressed genes to perform pathway analysis with Reactome and found that ERBB2 signaling, integrin cell-surface interactions and various metabolic processes were reduced in luminal-like breast tumors whereas interferon signaling, expression of glycoprotein tumor antigens termed T and Tn, and mitotic rates were increased in these same tumors. A complete list of the biological processes and signaling pathways unique to the luminal-like subtype tumors is shown in Table S7. The combination of these processes is unique to luminal-like subtype tumors; hence it would seem that luminal-like subtype tumors are biologically distinct from tumors representative of the other molecular subtypes.

## Discussion

Our analyses of nearly 1,600 publically available breast tumor gene expression profiles revealed that roughly 25% of these could not be molecularly classified in keeping with previous findings using a much smaller number of samples [1], [2], [3]. We found that all the previously unclassifiable breast tumors form a new molecular subtype, which we termed luminal-like. The latter subtype was also identified in an independent cohort of 295 breast tumors suggesting that this new subtype is reproducible. The global gene expression profiles of the luminal-like molecular subtype tumors are most closely related to those of the luminal A and luminal B subtypes, but differ from those of the luminal C subtype described previously [2]. The existence of the luminal-like subtype is also consistent with a report suggesting that unclassifiable samples are having transcript profiles similar to those of luminal tumors [21]. In keeping with the latter it is noteworthy that the luminal-like molecular subtype is characterized by high expression of ER and/or PR predictive gene signatures in 95.2% of these samples [15].

Whereas the gene expression profiles of the luminal-like subtype are related to those of the luminal A or B subtypes, the luminal-like subtype is nonetheless distinct. As shown by correlation coefficients of luminal-like samples to centroids of established molecular subtypes, transcripts expressed by only 25% or 28% of luminal-like subtype tumors were similar to those expressed by tumors of the luminal A or B subtype respectively (see Figure 2A). However, the level of similarity was low as all observed correlation coefficients were lower than 0.3. Tumors of the luminal-like subtype are also distinct from those of the luminal C subtype [2] because the luminal-like tumors did not express transcripts characteristic of luminal C subtype tumors.

Analysis of biological processes and signaling pathways inferred to be active in luminal-like tumors suggests that ERBB2 signaling, integrin cell-surface interactions and various metabolic processes are reduced whereas interferon signaling pathway activity is increased in these tumors compared to those of the other molecular subtypes. The biological processes and pathways characteristic of luminal-like tumors may be linked to patient survival. For example, high expression of integrin transcripts promotes tumor cell survival in various cancers [22], [23]. Additionally, it has been reported that high levels of integrin beta 2 (ITGB2) are related to poor outcome in leukemia [24]. Although the effects of high ITGB2 expression on patient outcome in breast cancer have not been studied, such effects were reported for another integrin (integrin alpha9 beta1), indicating the possibility that ITGB2 may be a marker of poor outcome in breast cancer patients [25].

We also found that luminal-like tumors are characterized by an increase in mitotic rates, which is reported to be correlated with poor clinical outcome [26]. Additionally, we found that luminal-like tumors are predicted to exhibit increased interferon alpha/beta signaling, as indicated by up-regulated IFITM1, IFIT1, ISG15, IFI27, and MX1 gene transcripts. Three of 5 genes (IFIT1, ISG15, IFI27) that comprise the DNA damage resistance signature (IRDS) are also highly expressed in luminal-like tumors, suggesting that these tumors might be resistant to DNA-damaging therapies [27]. On the other hand, down-regulation of ERBB2 signaling in luminal-like tumors might be linked to a better prognosis for patients who experienced such tumors [28], [29], [30]. Additionally, down-regulation of lipid and lipoprotein metabolism has also been shown to be linked to better prognosis because up-regulation of lipid metabolic enzymes, and those of cholesterol in particular, predict resistance to tamoxifen treatment [31]. Therefore, down-regulation of lipid and lipoprotein metabolism would predict sensitivity to tamoxifen.

These findings also point to the duality observed for the prognosis of patients who harbored luminal-like tumors: patients who experienced luminal-like subtype tumors are predicted to have a better outcome than those who had basal-like subtype tumors, but a worse outcome than those who had luminal A or normal-like subtype tumors. Importantly, we showed, that if luminal-like samples were forced to be classified into the established subtypes by using a correlation coefficient cutoff less than 0.3, then survival rates of the patients bearing these tumors do not reflect the distribution of the samples across these subtypes. More specifically, tumors from patients with poor outcome would not be classified into subtypes with known poor survival, whereas tumors from patients with good outcome would not necessarily be classified into subtypes with good survival rates. This finding indicates that the duality of prognosis in patients with luminal-like tumors is not an artifact, resulting from distribution of luminal-like tumors across the established subtypes.

In summary, patients who experienced tumors of the luminal-like subtype did not have either the best or the worst prognosis compared to patients who experienced tumors of one of the other 7 molecular subtypes. The latter may be explained by the various biological processes that are active in tumors of one or another molecular subtype. The luminal-like subtype seems to be stable and should aid efforts to identify therapies that target tumors of this molecular subtype.

## Supporting Information

Figure S1 **Hierarhical clustering of the standardized centroids of luminal-like and the other molecular subtypes.** Clustering was performed by using average linkage; columns were clustered by using Spearman distance, and rows – by using Euclidean distance.
(TIF)

Table S1 **710 intrinsic genes used for assigning molecular subtypes to 1,593 tumor samples.**
(XLS)

Table S2 **518 intrinsic genes used for assigning molecular subtypes to tumor samples from NKI cohort.**
(XLS)

Table S3 **Genes used for pathway analysis.** Event identifiers were obtained from Reactome analysis; p-values were adjusted by using Benjamini&Yekutieli method for FDR. Hierarchy of events, when present, was marked by “•”, their number corresponding to the level within the hierarchical tree of events (Pathway Browser at http://www.reactome.org/ReactomeGWT/entrypoint.html).
(XLS)

Table S4 **Molecular subtypes of the samples following classification with and without maximal correlation coefficient threshold of 0.3.**
(XLS)

Table S5 **Genes differentially expressed between Luminal-like and the other subtypes.** Differentially expressed genes were found from pair-wise comparisons between Luminal-like and the rest of the molecular subtypes. Total of 11,982 were used for the analyses.
(XLS)

Table S6 **Genes differentially expressed between Luminal-like and the other subtypes.** Differentially expressed genes were found from pair-wise comparisons between Luminal-like and the rest of the molecular subtypes. Total of 710 genes were used for the analyses.
(XLS)

Table S7 **Processes up- and down-regulated in samples belonging to Luminal-like subtype.** Event identifiers were obtained from Reactome analysis; p-values were adjusted by using Benjamini&Yekutieli method for FDR (see Methods). Hierarchy of events, when present, was marked by “•”, their number corresponding to the level within the hierarchical tree of events (Pathway Browser at http://www.reactome.org/ReactomeGWT/entrypoint.html).
(DOC)
