# Supplementary figures and images for: Identification of a Novel Luminal Molecular Subtype of Breast Cancer
Source: PLoS One. 2014 Jul 30;9(7):e103514. doi: 10.1371/journal.pone.0103514 (PMC4116208; doi:10.1371/journal.pone.0103514)

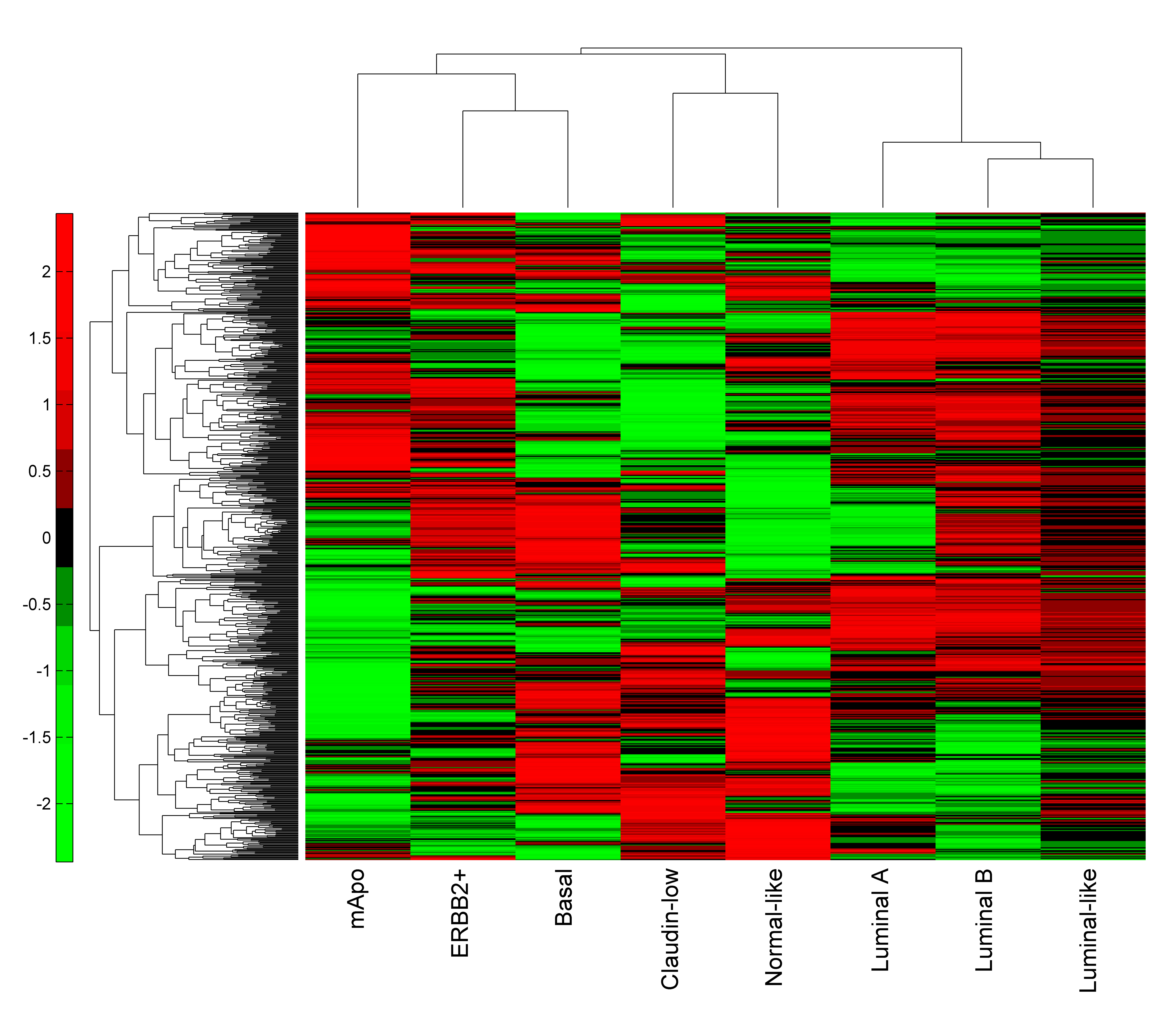

Supplement: Figure S1 — Hierarhical clustering of the standardized centroids of luminal-like and the other molecular subtypes. Clustering was performed by using average linkage; columns were clustered by using Spearman distance, and rows – by using Euclidean distance. (TIF) [file pone.0103514.s001.tif]
