# Supplementary material for: Identification of a Novel Luminal Molecular Subtype of Breast Cancer
Source: PLoS One. 2014 Jul 30;9(7):e103514. doi: 10.1371/journal.pone.0103514 (PMC4116208; doi:10.1371/journal.pone.0103514)
Supplement: Table S7 — Processes up- and down-regulated in samples belonging to Luminal-like subtype. Event identifiers were obtained from Reactome analysis; p-values were adjusted by using Benjamini&Yekutieli method for FDR (see Methods). Hierarchy of events, when present, was marked by “•”, their number corresponding to the level within the hierarchical tree of events (Pathway Browser at http://www.reactome.org/ReactomeGWT/entrypoint.html). (DOC) [file pone.0103514.s008.doc]

| **Name of this Event** | **Identifier of this Event** | **Total # of genes involved in this Event** | **# of genes mapping to the event** | **Genes mapping to the event** | **p-value**  **BY adjusted** |
| --- | --- | --- | --- | --- | --- |
| **Down-regulated processes** | | | | | |
| **Metabolism** | | | | | |
| Metabolism | REACT_111217 | 1083 | 47 | G0S2, CRAT, G6PD, ACADL, SRD5A1, GALE, KMO, BLVRB, CYP2J2, ACSM1, PNMT, ASS1, FMO2, BBOX1, PAPSS2, DHCR24, PFKP, KYNU, FAH, UGT2B28, ADH1B, ETFA, CAV1, LDHB, FAR2, MCCC1, ACSL1, GGT1, LPL, SCP2, PHGDH, AKR1D1, ACADM, QPRT, SC4MOL, PSMD3, HMGCS2, HPD, IDH1, FDFT1, GSTP1, FABP6, IDI1, NAMPT, IDH2, SDC1, DHCR7 | 1.09E-08 |
| Metabolism of lipids and lipoproteins | REACT_22258 | 315 | 19 | G0S2, CRAT, FAR2, ACADL, ACSL1, LPL, SRD5A1, SCP2, AKR1D1, ACADM, SC4MOL, HMGCS2, IDH1, DHCR24, FDFT1, FABP6, IDI1, SDC1, DHCR7 | 9.25E-05 |
| • Biological oxidations | REACT_13433 | 138 | 8 | FMO2, PAPSS2, GGT1, ACSM1, CYP2J2, GSTP1, ADH1B, UGT2B28 | 0.036 |
| • Cholesterol biosynthesis | REACT_9405 | 22 | 5 | SC4MOL, FDFT1, IDI1, DHCR7, DHCR24 | 0.001 |
| • Mitochondrial fatty acid beta-oxidation of unsaturated fatty acids | REACT_160 | 6 | 2 | ACADL, ACADM | 0.043 |
| • Peroxisomal lipid metabolism | REACT_16957 | 20 | 4 | SCP2, CRAT, FAR2, IDH1 | 0.012 |
| •• Regulation of Lipid Metabolism by Peroxisome proliferator-activated receptor alpha (PPARalpha) | REACT_19241 | 61 | 5 | G0S2, FDFT1, ACSL1, ACADM, HMGCS2 | 0.043 |
| ••• PPARA Activates Gene Expression | REACT_116145 | 42 | 5 | G0S2, FDFT1, ACSL1, ACADM, HMGCS2 | 0.021 |
| • Metabolism of amino acids and derivatives | REACT_13 | 191 | 10 | BBOX1, PSMD3, MCCC1, HPD, KMO, PNMT, ASS1, PHGDH, FAH, KYNU | 0.033 |
| **Signaling by SCF-KIT** | | | | | |
| Interaction of other adapter proteins with p-KIT | REACT_111192 | 6 | 2 | GRB7, KIT | 0.043 |
| **Platelet activation, signaling and aggregation** | | | | | |
| Exocytosis of platelet alpha granule contents | REACT_21351 | 47 | 5 | PROS1, CLU, FGG, SRGN, IGF1 | 0.030 |
| **Integrin cell-surface interactions** | | | | | |
| Interaction of integrin alphaDbeta2 with fibrin | REACT_13787 | 5 | 2 | FGG, ITGB2 | 0.036 |
| Interaction of integrin alphaXbeta2 with fibrin | REACT_13612 | 5 | 2 | FGG, ITGB2 | 0.036 |
| **Signaling by ERBB2** | | | | | |
| GRB7 events in ERBB2 signaling | REACT_115896 | 5 | 2 | GRB7, ERBB2 | 0.036 |
| GRB7 binds phosphorylated heterodimer of ERBB2 and ERBB3 | REACT_115703 | 5 | 2 | GRB7, ERBB2 | 0.036 |
| Up-regulated processes | | | | | |
| **Interferon signaling** | | | | | |
| Interferon alpha/beta signaling | REACT_25162 | 103 | 5 | IFITM1, IFIT1, ISG15, IFI27, MX1 | 0.012 |
| Expression of IFN-induced genes | REACT_25192 | 78 | 5 | IFITM1, IFIT1, ISG15, IFI27, MX1 | 0.006 |
| **Interferon signaling - Antiviral mechanism by IFN-stimulated genes** | | | | | |
| ISGylation of host proteins | REACT_115890 | 15 | 3 | IFIT1, ISG15, MX1 | 0.005 |
| Regulation of protein ISGylation by ISG15 deconjugating enzyme USP18 | REACT_115684 | 12 | 3 | IFIT1, ISG15, MX1 | 0.005 |
| **Post-translational protein modification** | | | | | |
| Addition of GalNAc to mucins to form the Tn antigen | REACT_115586 | 39 | 3 | MUC1, GALNT6, GALNT7 | 0.032 |
| **Regulation of mitotic cell cycle** | | | | | |
| Phosphorylation of Cdh1 by Cyclin B1:Cdc2 | REACT_6811 | 3 | 2 | CDH1, CCNB1 | 0.005 |
| Phosphorylation of Emi1 | REACT_6875 | 6 | 2 | CDH1, CCNB1 | 0.011 |
| Phosphorylation of the Emi1 DSGxxS degron by Cyclin B:Cdc2 | REACT_6891 | 5 | 2 | CDH1, CCNB1 | 0.009 |
